# Immunology of Vascularized Composite Allografts

**DOI:** 10.1155/2013/689071

**Published:** 2013-03-18

**Authors:** Gerald Brandacher, David H. Sachs, Angus W. Thomson

**Affiliations:** ^1^Department of Plastic and Reconstructive Surgery, Johns Hopkins University School of Medicine, Baltimore, MD 21205, USA; ^2^Transplantation Biology Research Center, Massachusetts General Hospital, Harvard Medical School, Boston, MA 02129, USA; ^3^Thomas E. Starzl Transplantation Institute, University of Pittsburgh, Pittsburgh, PA 15213, USA

Over the past decade, vascularized composite allotransplantation (VCA), such as hand and face transplantation, has become a clinical reality and a viable treatment option for those patients suffering from complex tissue injuries or defects not amenable to conventional reconstruction. Despite the fact that early and intermediate functional outcomes are highly encouraging rejection and the need for chronic immunosuppressive treatment continue to be the bane of VCA, preventing its broader clinical application.

A thorough understanding of the mechanisms underlying alloimmune responses in VCA is key to establish novel protocols for immunomodulation and tolerance induction after this type of transplant, without the need for long-term immunosuppression. Such advances would diminish the risks and favor the benefits for these non-life-saving but life-changing transplants.

This special issue is devoted to the immunology of vascularized composite allografts. The individual authors are leaders in the field, with extensive knowledge and expertise in this novel and emerging field of transplantation. The goal of this issue is to cover various specific aspects, as well as some of the immunological challenges related to VCA. The main focus is on tolerance strategies and how those apply to VCA.

Greater scrutiny is applied to immunosuppression-induced complications in VCA recipients compared to solid organ transplant patients since VCA is deemed life-enhancing rather than life-saving interventions. K. V. Ravindra et al. discuss the pressure to develop tolerance-inducing strategies in VCA.

D. A. Leonard et al. provide a comprehensive review of the use of mixed chimerism approaches for tolerance induction in the field of VCA with a particular emphasis on translational large animal (MGH miniature swine) protocols. The authors also provide an interesting overview of novel cellular therapies, such as regulatory T cells, regulatory dendritic cells, or mesenchymal stem cells as potential adjuvants to mixed hematopoietic chimerism in the development of tolerance induction protocols for clinical VCA.

M. Siemionow and A. Klimczak discuss the Cleveland Clinic research experience with chimerism-based experimental small animal models for tolerance induction and highlight in particular the complexity of immunomodulatory protocols in VCA as well as their relevance and applicability for clinical practice.

W.-C. Huang et al. review approaches to improve the safety of tolerance inducing regimes currently applied to VCA and discuss immune monitoring and tolerance assays that are critically required prior to translating such protocols in the clinic.

Y.-R. Kuo et al. summarize the current understanding of immunomodulation achieved by mesenchymal stem cell (MSC) therapy and provide a possible outline for its future clinical application in VCA.

R. Starzl et al. provide a historically oriented discussion of cross-disciplinary approaches to develop novel points of view for some of the most challenging immunological problems in VCA, particularly the early diagnosis and assessment of rejection. Some of these approaches and methods lie at the intersection of medicine, immunology, mathematics, and computer science. By leveraging the strengths and capabilities of each discipline to solve problems that have been resistant to analysis in another, more rapid progress can be made in delivering novel and clinically relevant findings, diagnostics, or therapeutic agents.

T. Hautz and colleagues review the key players and molecular events of skin inflammation and discuss new therapies originally developed in solid organ transplantation with particular emphasis on skin allograft rejection and how such therapies could relate to VCA. This is of interest since the mechanisms and dynamics of acute and chronic skin allograft rejection are incompletely understood and remain the subject of numerous ongoing trials aimed at better understanding of the underlying pathophysiology and at novel and targeted drug development.

J. T. Schnider et al. provide an overview of graft-targeted and delivered site-specific immunosuppression, which is uniquely well suited for VCA due to their direct accessibility to such interventions. The authors highlight the fact that site-specific therapeutic effects and efficacy of systemically active agents may enable optimal dosing, frequency, and duration of overall immunosuppression in VCA, with minimization or elimination of long-term drug-related toxicity.

C. G. Wallace et al. show that intrajejunal treatment with donor splenocytes could render recipients immunologically hyporesponsive in a donor-specific manner *in vitro*. When the regimen was tested in a rodent setting *in vivo*, VCA rejection was delayed but did not result in immunosuppressive drug-free tolerance. These encouraging data warrant further investigation to assess the exact role and mechanisms of intrajejunal treatment as a low-risk adjunct to prevent VCA rejection.

Q. Liu et al. provide interesting evidence that a short-course of combined antilymphocyte serum and Cyclosporine A treatment enables indefinite VCA survival, which induces secondary donor-specific skin and heart allograft tolerance despite the loss of peripheral chimerism. These findings open up new perspectives for the role of VCA in the induction and maintenance of tolerance to solid organ transplants.

We hope that this series of articles will stimulate continuing efforts to better understand the unique immunological features of VCA compared to solid organ transplants, and the development of novel strategies to induce tolerance.

